# Data of interaction of supported ionic liquids phases onto copper nanoparticles: A density functional theory study

**DOI:** 10.1016/j.dib.2020.106562

**Published:** 2020-11-23

**Authors:** Kerry Wrighton-Araneda, Cristián Valdebenito, Gabriel Abarca, Diego Cortés-Arriagada

**Affiliations:** a Programa Institucional de Fomento a la Investigación, Desarrollo e Innovación. Universidad Tecnológica Metropolitana, Ignacio Valdivieso 2409, P.O. Box, San Joaquín, Santiago 8940577, Chile; b Universidad Bernardo O'Higgins, Escuela de Obstetricia y Puericultura, Centro Integrativo de Biología y Química Aplicada (CIBQA), Santiago 8370993, Chile

**Keywords:** DFT calculations, Ionic liquids, Adsorption energy, Nanoparticles

## Abstract

This work contains data on the computational, structural, and electronic characterization of supported ionic liquids phases anchored to copper nanoparticles using Density Functional theory calculations. The data supplement the paper “Interaction of supported ionic liquids phases onto copper nanoparticles: A Density Functional Theory study” [1], based on the adsorption of ionic liquid onto a Cu nanoparticle is analyzed from a chemical and physical point of view. The chemical analysis is based on Atoms in Molecule theory (AIM) and allows us to differentiate the chemical binding nature between ionic liquid and copper nanoparticle. On the other hand, the energy decomposition analysis based on absolutely localized molecular orbital (ALMO-EDA) describes the physical contributions that govern the interaction between ionic liquid and the copper nanoparticles. Herein, detailed and extended information in the synthesis and computational characterization are presented.

## Specifications Table

**Table:** 

Subject	Chemistry
Specific subject area	Physical and Theoretical chemistry
Type of data	Graph, Fig, and Image.
How data were acquired	Computational data was acquired using Density functional theory calculations for structures optimization. Adsorption energy was calculated using electronic energy. Thermodynamic parameters for the adsorption process were calculated from the vibrational analysis. Energy decomposition analysis was employed to determine physical contribution. The nature of chemical bonding was studied using Atom in Molecules analysis (AIM). Experimental data were acquired using X-ray photoelectron spectroscopy (XPS) measurements were performed using a Kratos AXIS Ultra DLD instrument. The chamber pressure during the measurements was 5 × 10^−9^ Torr. Wide energy range survey scans were collected at pass energy of 80 eV in hybrid slot lens mode and a step size of 0.5 eV. High-resolution data on the C 1 s, N 1 s, and F 1 s photoelectron peaks were collected at pass energy 20 eV over energy ranges suitable for each peak, and collection times of 5 min, step sizes of 0.1 eV. The charge neutralizer filament was used to prevent the sample from charging over the irradiated area. The X-ray source was a monochromated Al K_α_ emission, run at 10 mA and 12 kV (120 W). The energy range for each ‘pass energy’ (resolution) was calibrated using the Kratos Cu 2p_3/2_, Ag 3d_5/2,_ and Au 4f_7/2_ three-point calibration method. The data were charge corrected to the reference carbon adventitious signal at 284.8 eV X-ray photoelectron spectroscopy.
Data format	Raw data and analyzed
Parameters for data collection	Computational data: The DFT calculations were developed considering standard convergence criteria for self-consistent field and geometry optimization procedures. Experimental data: XPS measurements were carried out using a Kratos AXIS Ultra DLD instrument. High-resolution XPS data on the C 1 s, N 1 s, and F 1 s photoelectron peaks were collected at pass energy 20 eV over energy ranges suitable for each peak, collection times of 5 min, and step sizes of 0.1 eV.
Description of data collection	Computational data: the data were collected from DFT calculations using visualization tools as VIM, Geany, and Chemcraft programs. Further wavefunction analyses were developed using the Multiwfn3.6 program. Experimental data: XPS provided insight into the interactions between surface Cu NP and the ionic liquids. The transmission function was calibrated using a clean gold sample method for all lens modes and the Kratos transmission generator software within Vision II. The data were processed with CASAXPS (Version 2.3.17).
Data source location	Computational Data: Programa Institucional de Fomento a la Investigación, Desarrollo e Innovación. Universidad Tecnológica Metropolitana. Ignacio Valdivieso 2409, P.O. Box 8,940,577, San Joaquín, Santiago, Chile. Experimental Data: Universidad Bernardo O'Higgins, Escuela de Obstetricia y Puericultura, Centro Integrativo de Biología y Química Aplicada (CIBQA), Santiago, 8,370,993, Chile.
Data accessibility	Data are available within this article Repository name: Mendeley Data Data identification number: 1 Direct URL to data: http://dx.doi.org/10.17632/zr3vf3bxpk.1 https://data.mendeley.com/datasets/zr3vf3bxpk/1 DOI:10.17632/zr3vf3bxpk.1
Related research article	*K. Wrighton-Araneda, C. Valdebenito, M.B. Camarada, G. Abarca, D. Cortés-Arriagada. Interaction of supported ionic liquids phases onto copper nanoparticles: A DFT study. Journal of Molecular Liquids 310 (2020) 113,089*. DOI:10.1016/j.molliq.2020.113089

## Value of the Data

• The new Cu@(X)SILPs synthesized systems open a new route of triazolium based SILPs for catalysis and technological applications.
• The work explores the chemical and physical properties involved in the interaction of a new family of triazolium based SILP onto Cu NPs.
• The determination of the physical contributions involved in Cu@(X)SILPs complexes provide a deeper understanding of the stabilization and adsorption phenomena.

## 1 Data Description

**Fig. 1:**
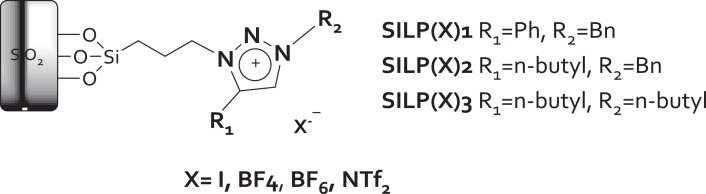
Synthetic route for obtention of SILP(X)1–3. Modified from [1].

For this work, we designed a series of new triazolium-based supported ionic liquids (SILPs), decorated with Cu NP (Cu@SILPs). The triazoles moieties were functionalized using copper-catalyzed azide-alkyne cycloaddition. Three triazolium cations (T1^+^, T2^+^, and T3^+^) and four anions (*I*^−^, BF_4_^−^, PF_6_^−^, and NTf_2_^−^) were considered to form the Cu@SILPs complexes. XPS and computational analysis gave mechanistic insights into the Cu NP stabilization pathways, where the anion adsorption onto Cu NP is favored compared to the cation adsorption. The stronger adsorption is observed for Cu@(I)SILP1 complex, which presents the more electron-rich triazole and the higher adsorption value of SILP onto Cu surface (5.18 eV). Computational studies of the adsorption of SILP onto Cu NPs allow evaluating the chemical and physical properties that govern these complexes stability. The schematic representation of Cu@(X)SILPs complexes is displayed in Fig 1.

The Cu@SILPs complexes stability was characterized by the adsorption energy (*E_ads_*) (Eq. (1)), where the *E_Cu_, E_cat_, E_ani_,* and *E_Cu@SILP_* correspond to the total energy of Cu_55_, ionic liquid triazolium cation, ionic liquid anion, and the whole Cu@SILP systems. A more positive value of *E_ads_* indicates a higher stabilization. The standard counterpoise method of *Boys* and *Bernardi* was implemented to correct the energy basis set superposition error (BSSE) [2].

From a physical viewpoint, the adsorption energy can be divided into two terms (Eq. (2)): preparation energy (Δ*E_PREP_*) and interaction energy (Δ*E_INT_*). The energy decomposition analysis based on absolutely localized molecular orbital (ALMO-EDA), implemented in Q-Chem 5.2 computational package [3,4], allows separating the adsorption energy into physically meaningful terms [5]. According to this scheme, the *E_ads_* of a three-fragment A-B-C complex can be decomposed in their physical terms (Eq. (3)):

(1) $$E_{ads}=E_{Cu}+E_{cat}+E_{ani}-E_{Cu@SILP}$$

(2) $$-E_{ads}=\Delta E_{PREP}+\Delta E_{INT}$$

(3) $$-E_{ads}=\Delta E_{PREP}+\Delta E_{POL}+\Delta E_{CT}+\Delta E_{ELEC}+\Delta E_{PAULI}+\Delta E_{DISP}$$

**Fig. 2:**
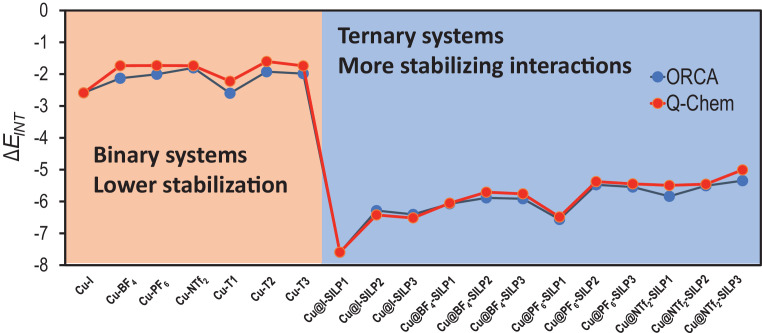
Comparative plot trends of the interaction energy calculated using ORCA and Q-Chem for the binary and ternary systems based on the Cu@(X)SILPs, where *X* = *I*, BF_4_, PF_6_, and NTf_2_. Modified from [1].

Where Δ*E_POL_*, Δ*E_CT_*, and Δ*E_ELEC_* correspond to Polarization, Charge-Transfer, and Electrostatic energies as the stabilizing physical contributions, while Δ*E_PAULI_* and Δ*E_PREP_* energy are the destabilizing physical contributions. To verify ORCA consistency with Q-Chem5.2 (ALMO-EDA) calculations, the Δ*E_INT_* was employed as a comparative case (Fig. 2). Due to the tendency and values calculated between both programs are consistent, further studies were performed. Fig. 2 displays the comparison between **Δ*E*_INT_** calculated using Q-chem and ORCA to validate both computational programs transferability.

The method to evaluate the interaction includes using two groups of system, the binary and ternary systems. Binary systems are formed by Cu NP bonded to an anion or cation, while ternary systems are formed by Cu NP, the anion, and the cation of the ionic liquid. The binary systems present a lower number of specific interactions, while ternary shows a full set of specific interactions. Therefore, the Δ*E_INT_* of binary is less stabilized (Fig. 2). This methodology evaluates the stabilization as a process of successive steps.

The thermodynamic function can be calculated from full relaxed structures based on the vibrational analysis. This analysis is carried out considering the pressure of 1 atm and temperature of 298.15 K. The thermodynamic functions ΔG, ΔH, and TΔS were calculated for the adsorption process, according to *ΔG_ads_=ΔH_ads_-TΔS_ad_*_s_. The thermodynamic functions indicate that reaction occurs spontaneously (*ΔG_ads_<*0) thanks to enthalpic contributions since the thermo-entropic function does not support the spontaneity of the reaction showing positive values of *-TΔS_ad_*_s_.

**Fig. 3:**
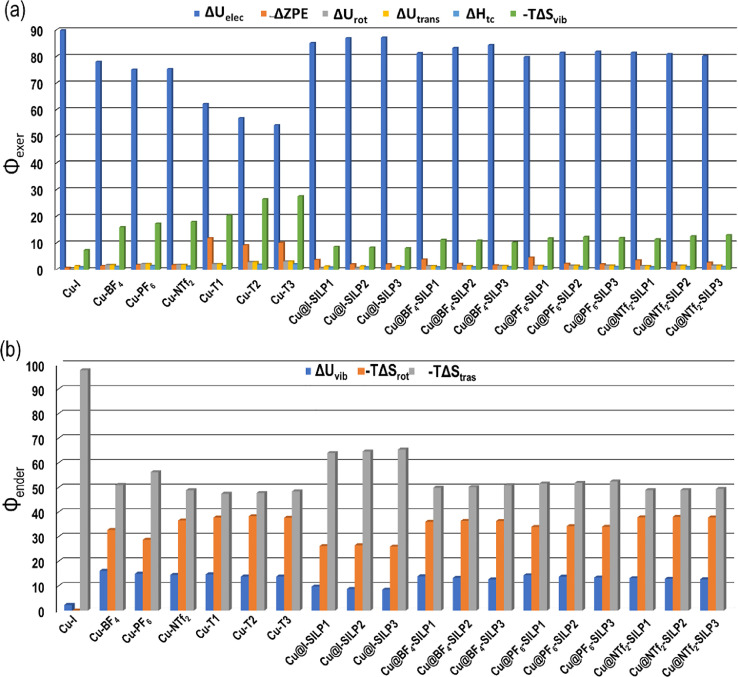
Percentual (a) exergonic contributions (Φ_exer_) and (b) endergonic contributions (Φ_ender_) for the binary and ternary systems based on the Cu@(X)SILPs, where *X* = *I*, BF_4_, PF_6_, and NTf_2_.

From the thermodynamic statistic point of view, each parameter can be divided according to the partition function. Thus, *U=U_elec_+ZPE+U_vib_+U_rot_+U_trans_*, where *U_elec_* is the electron energy, *ZPE* is the zero-point energy, *U_vib_* is the vibrational inner energy, *U_rot_* is the rotational inner energy, and *U_trans_* is the translational energy. Then, enthalpy is expressed as *H* = *U*+*H_corr_; H_corr_* is the thermal correction to enthalpy. The entropy is also divided by the partition function in the following parts: *S*= *S_elec_+S_vib_+S_rot_+S_trans_*, where *S_elec_, S_vib_, S_rot_*, and *S_trans_* are the electronic, vibrational, rotational, and translation entropy terms, being S_elec_=0 because there is no change in the multiplicity during the adsorption reaction. Thus, considering that the partition function was decomposed thermodynamically, the different contributions can affect the adsorption's spontaneity. Fig. 3 summarizes the percentual contributions of each partition function for the studied thermodynamic functions. The endergonic contribution (Δ*G* > 0) decreases the spontaneity, while the exergonic contributions (Δ*G* < 0), support the spontaneity of the adsorption reaction. Exergonic contributions correspond to Δ*U_elec_*, ΔZPE, Δ*U_rot_*, Δ*U_trans_, H_corr_*, and -*T*Δ*S_vib_*, and the endergonic contribution correspond to Δ*U_vib_*, -*T*Δ*S_rot_*, and -*T*Δ*S_trans_*. The exergonic component is dominated by the Δ*U_elec,_* which is the Eads negative, while for endergonic contribution is -*T*Δ*S_trans_* (Fig. 3a). Interestingly, the exergonic adsorption process showed a higher contribution of Δ*U_elec_* for the anion complexes, where the Cu-I^–^ complex presents 90% of Δ*U_elec_*, while the Cu-PF_6_^–^ and Cu-NTf_2_^–^ complexes showed contributions of 75% of Δ*U_elec_*. Another exergonic contribution corresponds to the increase of vibrational entropy showing the highest value for Cu-NTf_2_^–^ complex (18%) and the lowest for Cu-I^–^ (7%). For the endergonic terms, Cu-I^–^ complex exhibits the highest contribution of 98%, associated with a decreasing of the translation entropy, whereas the Cu-NTf_2_^−^ complex shows the lowest contribution reaching 49%.

For cations, the highest exergonic contribution was observed by the Δ*U_elec_* term in the Cu-T1^+^ complex with 62%, while the lowest value was 54% for Cu-T3^+^. The endergonic contribution of each cation has the same contributions for Δ*U_vib_*, -*T*Δ*S_rot_*, and -*T*Δ*S_trans_* with values of 14, 38, and 48%, respectively. For the Cu@(X)SILP complexes, the Δ*U_elec_* and -*T*Δ*S_vib,_* are the most critical terms, where the values are ranged between 80 and 87% and 8–13%, respectively. A similar landscape is observed for an endergonic term where the *T*Δ*S_rot_*, and -*T*Δ*S_trans_* displays higher contributions with values of 26–38% and 49–65%, respectively.

The detailed information of the carried about thermodynamic analyses can be revised in the attached data linked to this work (files “Thermodynamic-decomposition-Analysis-CuSILP.xlsx” and “Thermodynamic-functions-cusilp.xlsx”). Formulas and values are included in the cited files.

The structural analysis of the intermolecular interaction of Cu-anion, Cu-cation, and Cu@SILP1–3 complexes can be described using Atom in Molecules analysis evaluating the electron density at the bond critical points (BCPs). The atom in molecules (AIM) studies were developed considering the following critical point searching parameter: Maximal iterations of 1000; a scale factor of step-size of 0.50; criteria for electron density gradient-norm convergence of 1.0 × 10^−6^; criteria for displacement convergence: 1.0 × 10^−7^; minimal distance between Critical Points of 0.03 Bohr. Skip search if the distance between atoms is longer than the sum of their van der Waals radius multiplied by 1.80; and the criteria for determining if the Hessian matrix is singular of 1.0 × 10^−50^. Detailed information for the AIM evaluation can be checked in the data set included in this article; specifically, in the directory AIM-Summary. The files can be visualized with any text editor; however, the authors recommend Geany and content some modifications to obtain the Poincare-Hopf condition required for these analyses (DOI: http://dx.doi.org/10.17632/zr3vf3bxpk.1).

**Fig. 4:**
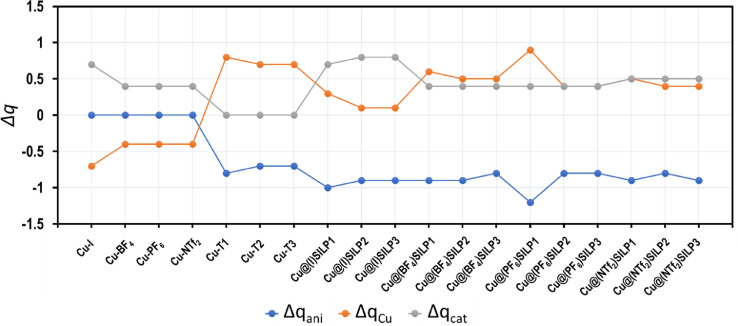
Plot of fragmental charges employing the population model CM5 developed by Truhlar for the binary and ternary systems based on the Cu@(X)SILPs, where *X* = *I*, BF_4_, PF_6_, and NTf_2_.

The donor-acceptor interactions were evaluated using the fragmental charges (Δ*q*). Fig. 4 summarized the obtained results for the complex fragments, including Cu_55_, anions, and cations. Detailed information about the fragmental charge calculation can be revisited in the fragmental-Charge-CM5.xlsx file included in the Data set of these articles. The file can be used as payroll for other studies since it contains the formulae for the fragment calculation.

**Fig. 5:**
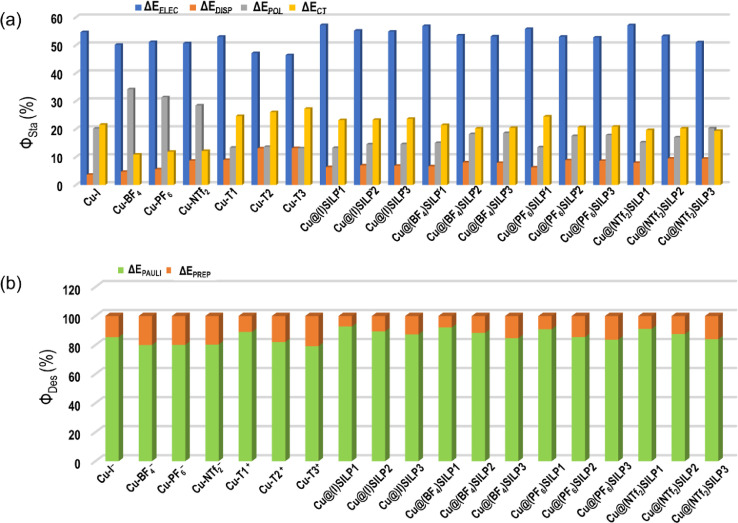
Relative percentual contributions to the (a) stabilizing and (b) destabilizing energy binary and ternary systems based on the Cu@(X)SILPs, where *X* = *I*, BF_4_, PF_6_, and NTf_2_. Calculations based on ALMO-EDA method.

The ALMO-EDA results for stabilizing and destabilizing terms are presented (Fig. 5). The destabilizing terms for the studied complexes are displayed graphically in Fig. 5b, showing the dominance of ΔE_PAULI_ in destabilizing terms.

**Fig. 6:**
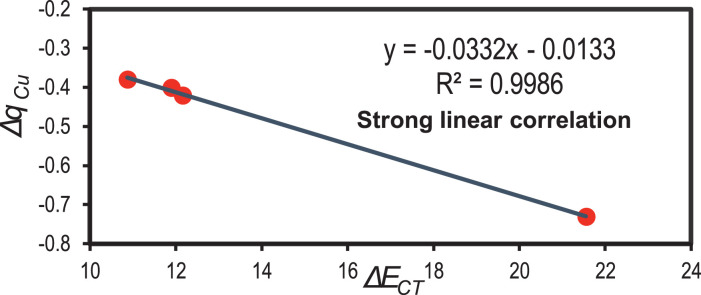
Plot Δ*q_Cu_* vs Δ*E_CT_* for anionic binary systems: Cu-I^–^, Cu-BF_4_^–^, Cu-PF_6_^–^, and Cu-NTf_2_^–^. Linear correlation is described inset. Modified from [1].

For the anionic complexes, the correlation between Δ*q_Cu_* and Δ*E_CT_* is presented in Fig. 6, where it is possible to observe that fragmental charge is linearly correlated to charge transfer energy.

**Fig. 7:**
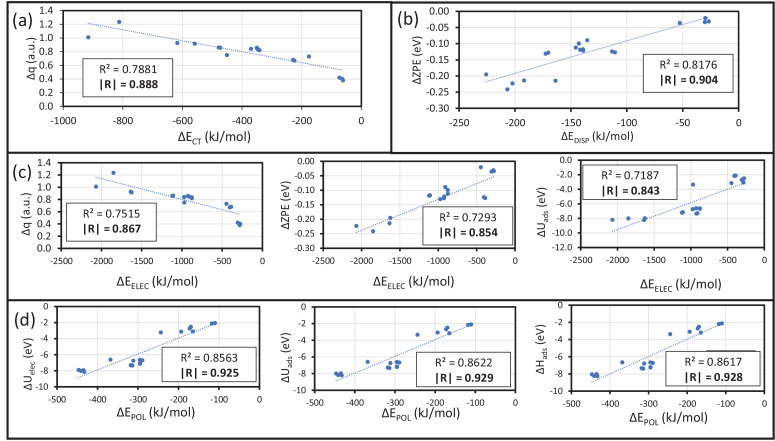
Correlation plot for the (a) ΔE_CT_, (b) ΔE_DISP_, (c) ΔE_ELEC_, and (d) ΔE_POL_ stabilizing terms. Modified from [1].

Other properties are related to the energy decomposition by the ALMO-EDA method. A correlation analysis was carried to calculate the following properties: ionization potential (IP), electron affinity (EA), electronic chemical potential (μ), electron hardness (η), electrophilicity (ω), dipolar moment (δ), maximum electron transfer (Δ*q*), and the thermodynamic properties obtained from vibrational analysis such as Δ*U_ads_*, Δ*H_ads_*, -*T*Δ*S_ads_*, Δ*G_ads_* including its electronic, vibrational, rotational and translational contributions. For this purpose, the statistical analysis was developed using the linear regression procedure using an Excel program. The correlation plots are displayed in (Fig. 7). In the case of Δ*E_CT_,* the higher correlation parameter is Δ*q,* which is intrinsically related to the charge transfer phenomena and the electron flow between fragments (Fig. 7a). Secondly, Δ*E_DISP_* presents several high contributions, but the more correlated is the Δ*ZPE* with a value of 0.904 (Fig. 7b). However, other significant contributions are those related to δ since dispersion interaction also occurs from low weak electrostatic interaction with dipoles. For Δ*E_ELEC_,* the stronger correlations are Δ*q*, Δ*ZPE*, and Δ*U_tot_* with values of 0.867, 0.854, and 0.843 (Fig. 7c), respectively. This behavior can be explained, considering that the electron transfer contributes to the permanent coulombic interactions since Cu_55_ turns on a charged fragment that interacts with both anion and cation. The Δ*ZPE* and Δ*U_tot_* are relevant terms in the electronic energy of reaction, having a close relation to the higher contribution of Δ*E_ELEC_*. Finally, Δ*E_POL_*, the thermodynamic parameter without BSSE corrections (Fig. 7d), that is, Δ*U_elec_*, Δ*U_ads_*, and Δ*H_ads_* display strong correlations with 0.925, 0.929 and 0.928, respectively. The mentioned thermodynamic functions contain the stabilization energy mainly Δ*U_elec_* as a higher contribution indicating that the internal electronic contribution is intrinsically involved in polarization phenomena. Correlation analyses of all the before mentioned properties are displayed in the file “Correlation-Data.xlsx”; the detailed information of the correlation factor method and subsequent analysis are incorporated in the Data Set.

**Fig. 8:**
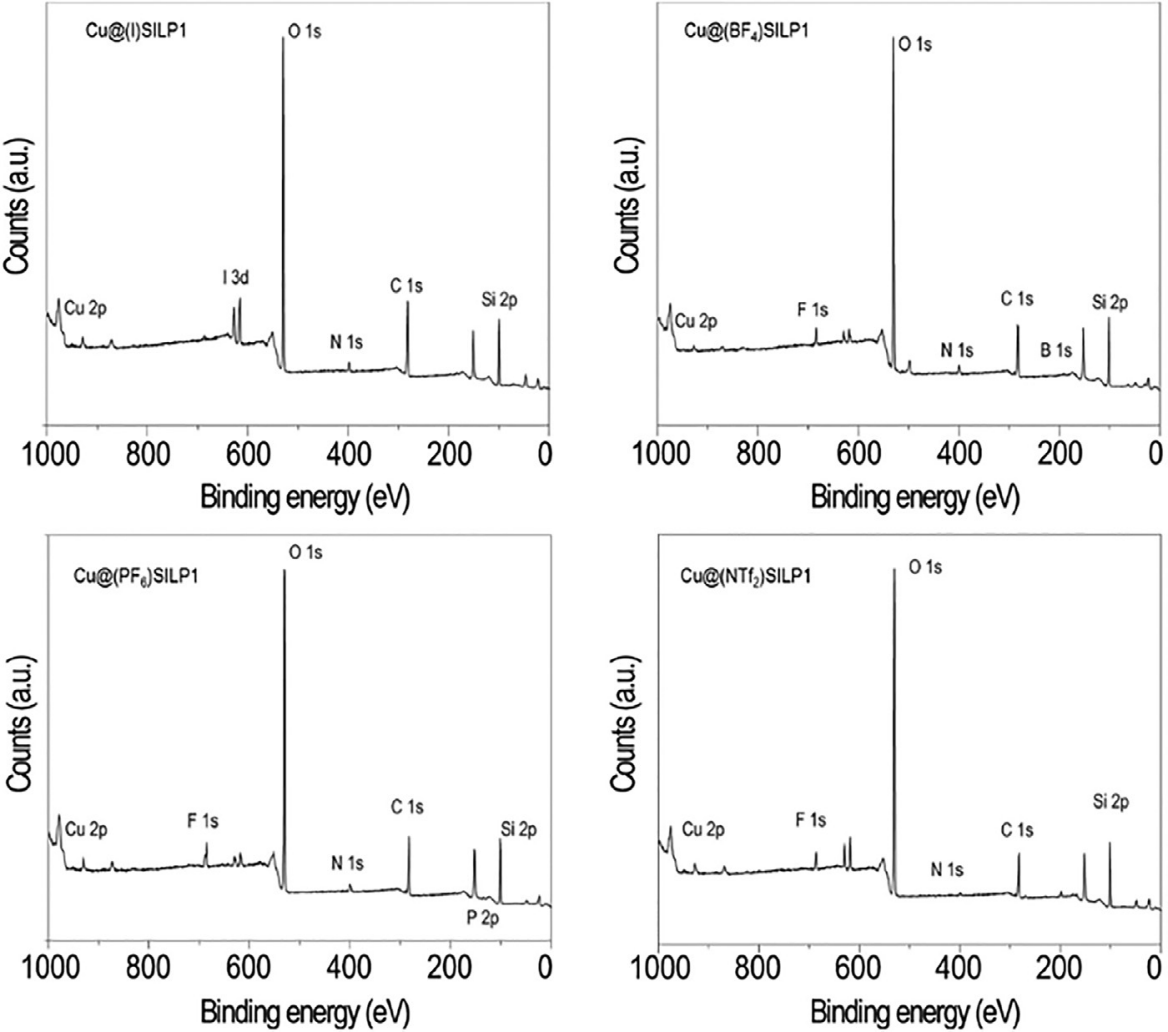
Wide range XPS spectra of Cu@(I)SILP1, Cu@(BF_4_)SILP1, Cu@(PF_6_)SILP1, and Cu@(NTf_2_)SILP1. Modified from [1].

Fig. 8 displays the XPS spectra of the Cu@SILP1 complexes with the respective anions.

Detailed information of the XPS spectra and raw spectra can be found in the file called “RAw Data in brief.opj” included in the Data Set attached to this work.

## 2 Experimental Design, Materials and Methods

### 2.1 Materials

Iodobenzene, 3-iodopropyltrimethoxysilane, benzyl bromide, phenylacetylene, NaN_3_, CuCl_2_×2H_2_O, CuI, NaBH_4_, MgSO_4_, NaBF_4_, KPF_6_, and LiNTf_2_, and BMI.BF_4_ were purchased from Sigma Aldrich. All chemicals were used without further purification, except for DCM, toluene, MeOH, acetonitrile, Et_2_O, and EtOAc, which were purified by standard procedures [6].

### 2.2 Synthesis of 1,2,3-Triazolium derivatives

Alkyne (5 mmol, 1.0 eq.), alkyl halide (1.0 eq.), and NaN_3_ (1.3 eq.) were loaded into a 25 mL round-bottom flask. Then [CuI(PPh_3_)_3_] (0.05 mol%) was added and dissolved in water (5 mL). The reaction was stirred for 4 h at room temperature, and the progress of the reaction was monitored using thin-layer chromatography (TLC). After its completion, the reaction mixture was filtered, and the residue was dissolved in DCM. The combined organic layer was later concentrated in a vacuum to yield the corresponding triazoles.

### 2.3 Synthesis of SILP(X) 1–3

SiO_2_–I (5 g) was suspended in dry acetonitrile (30 mL), with stoichiometric amounts of triazoles1–3 and refluxed during 24 h. Then, the particles were collected by filtration and repeatedly washed with petroleum ether, and then dried off under vacuum at 110 °C for 4 h to obtain SILP1–3 powder. Based on the amount of IL on the SILP(I)1–3 supports, an excess (1.2 eq.) of NaBF_4_, KPF_6_, and LiNTf_2_ salts were dissolved in deionized water (25 mL) and added to the SILP(I)1–3 (1.0 g) in order to exchange the anions. The suspensions were vigorously stirred for 48 h. The mixtures were washed, centrifuged, and dried to yield the supports SILP(BF_4_)1–3, SILP(PF_6_)1–3, and SILP(NTf_2_)1–3.

### 2.4 Synthesis of SiO_2_-I

Initially, 200 g mesh silica gel was soaked in 30% HCl overnight to hydrolyze its surface. Next, activated silica gel (100 g) was suspended in dry toluene (30 mL) in a round-bottom flask equipped with a reflux condenser under nitrogen. While being stirred, 3-iodopropyltrimethoxysilane (0.05 M) was added dropwise. The suspension was refluxed for 72 h. After cooling, the solid was collected by filtration and exhaustively washed by Soxhlet extraction with ethanol and water, and then dried under reduced pressure to yield iodopropyl–silica gel (SiO_2_–I).

### 2.5 Synthesis of Cu@(x)SILP1–3

A solution of CuCl_2_×2H_2_O (0.25 mmol) and MeOH (20 mL) was added to SILP(X)1–3 (100 mg) under constant stirring at room temperature for 30 min. A solution of NaBH_4_ (5 mmol) dissolved in MeOH (3 mL) was added to the reaction mixture dropwise. The reaction mixture turned black due to the formation of Cu NPs that were washed with MeOH (3 × 10 mL) and Et_2_O (3 × 10 mL). Subsequently, the samples were isolated by centrifugation (4500 rpm) and dried under reduced pressure.

### 2.6 X-ray photoelectron spectroscopy

XPS Experiment was collected by powder sample that was mounted on double-sided tape (Sellotape) and pressed to ensure proper coverage of the tape with the powder. X-ray Photoelectron Spectroscopy (XPS) measurements were performed using a Kratos AXIS Ultra DLD instrument. The chamber pressure during the measurements was 5 × 10^−9^ Torr. Wide energy range survey scans were collected at pass energy of 80 eV in hybrid slot lens mode and a step size of 0.5 eV. High-resolution data on the C 1 s, N 1 s, and F 1 s photoelectron peaks were collected at pass energy 20 eV over energy ranges suitable for each peak, and collection times of 5 min, step sizes of 0.1 eV. The charge neutralizer filament was used to prevent the sample from charging over the irradiated area. The X-ray source was a monochromated Al K_α_ emission, run at 10 mA and 12 kV (120 W). The energy range for each ‘pass energy’ (resolution) was calibrated using the Kratos Cu 2p_3/2_, Ag 3d_5/2,_ and Au 4f_7/2_ three-point calibration method. The data were charge corrected to the reference carbon adventitious signal at 284.8 eV. The transmission function was calibrated using a clean gold sample method for all lens modes and the Kratos transmission generator software within Vision II. The data were processed with CASAXPS (Version 2.3.17).

### 2.7 ALMO-EDA calculations

Calculations were calculated using Q-Chem 5.2 computational package [3,4]. For energy decomposition analysis based on absolutely localized molecular orbitals (ALMO-EDA), the following parameters were considered: Self-consistent Field algorithm corresponds to a mixture of DIIS and Geometrical Direct Minimization using an energy convergence criterion of 1 × 10^−6^ Hartree with no symmetry restriction. Stoll Fragmentation method employing Roothaan-step and exact SCF correction methods after the locally-projected equations. The basis set superposition error (BSSE) and dispersion D3BJ corrections were included.

### 2.8 AIM analysis

The atom in molecules studies (AIM) were developed using the Multiwfn3.6 program [7] and its routine of Topology analysis (option 2). These calculations were carried out considering the following critical point searching parameter: Maximal iterations of 1000; a scale factor of step-size of 0.50; criteria for electron density gradient-norm convergence of 1.0 × 10^−6^; criteria for displacement convergence: 1.0 × 10^−7^; minimal distance between Critical Points of 0.03 Bohr. Skip search if the distance between atoms is longer than the sum of their van der Waals radius multiplied by 1.80; and the criteria for determining if the Hessian matrix is singular of 1.0 × 10^−50^.

### 2.9 Fragmental charge calculation

Fragmental charges were calculated using the Charge Model 5 population analysis [8], which is implemented in the Multiwfn3.6 program [7] in the routine of population analysis and atomic charges (option 7).

## CRediT Author Statement

**Kerry Wrighton-Araneda:** Conceptualization, Formal analysis, Investigation, Methodology, Software, Validation, Visualization, Writing - original draft, Writing - review & editing. **Cristián Valdebenito:** Investigation, Writing - original draft, Visualization, Validation. **Gabriel Abarca:** Investigation, Conceptualization, Funding acquisition, Investigation, Methodology, Project administration, Resources, Software, Supervision, Writing - review & editing, Data curation. **Diego Cortés-Arriagada:** Investigation, Conceptualization, Funding acquisition, Investigation, Methodology, Project administration, Resources, Software, Supervision, Writing - review & editing, Data curation.

## Declaration of Competing Interest

The authors declare that they have no known competing for financial interests or personal relationships which have, or could be perceived to have, influenced the work reported in this article.
